# Patient safety in chiropractic teaching programs: a mixed methods study

**DOI:** 10.1186/s12998-020-00339-0

**Published:** 2020-09-18

**Authors:** Katherine A. Pohlman, Stacie A. Salsbury, Martha Funabashi, Michelle M. Holmes, Silvano Mior

**Affiliations:** 1 Parker University, 2540 Walnut Hill Lane, Dallas, TX 75229 USA; 2 Palmer Center for Chiropractic Research, Palmer College of Chiropractic, 1000 Brady St, Davenport, IA 52803 USA; 3 Canadian Memorial Chiropractic College, 6100 Leslie Street, Toronto, ON M2H 3J1 Canada; 4 AECC University College, Parkwood Campus, Parkwood Rd, Bournemouth, BH5 2DF UK

**Keywords:** Patient safety, Education, Chiropractic, Mixed methods, Quality improvement

## Abstract

**Background:**

Patient safety research has lagged within academic settings, including chiropractic teaching institutions. To develop a robust patient safety culture, the Institute of Medicine emphasized the need for employee’s attitudes to be understanding and positive. To initiate the assessment of the current culture and future needs, this study evaluated patient safety attitudes among chiropractic teaching clinic stakeholders (supervising clinicians, student interns, and administrative staff) and compared their standardized survey scores to established medical survey databases.

**Methods:**

We conducted a cross-sectional, mixed methods survey design with quantitative analytic priority. Chiropractic interns, clinical faculty, and clinic staff of 5 international chiropractic educational programs completed a modified version of the Agency for Healthcare Research and Quality (AHRQ) Patient Safety Culture for Medical Offices Survey with open-ended comment fields between 2014 and 2016. Composite means of positive responses were calculated and compared to patient safety, quality of care, and overall self-ratings benchmarks from Canadian providers and academic settings in the AHRQ database. Qualitative responses were thematically categorized for a convergent analysis of quantitative results for the chiropractic sample.

**Results:**

Chiropractic survey response rate was 45.3% (*n* = 645). Quantitative survey results indicated moderate scores and ranges (57–85%) on all patient safety dimensions for the chiropractic samples. Academic medicine and chiropractic providers’ benchmarks scored higher positive responses than chiropractic teaching clinics on most quantitative dimensions, except for work pressure/pace. Teamwork, organizational learning, and patient tracking/follow-up were the most positively endorsed quantitative dimensions, with communication, staff training, office standardization, and leadership support considered areas for improvement in both settings. Qualitative responses for the chiropractic clinics identified a need for open communication; additional staff training and student involvement in creating safety cultures; standardization of office processes including information exchange, scheduling, and equipment maintenance; and leadership support that focused on decreasing work pressure/pace and setting safety priorities.

**Conclusion:**

As the first report of patient safety attitudes from stakeholders in chiropractic teaching clinics, specific areas of improvement were identified. Chiropractic teaching programs might consider incorporating these and related patient safety concepts into their formal curricula. Mixed methods approach offers teaching clinics opportunities to assess stakeholders’ insights and enhance safe delivery of chiropractic care.

## Introduction

The Institute of Medicine (IOM) report, *To Err Is Human,* raised awareness about medical errors and challenged the healthcare community to develop a culture of patient safety [1]. Hospital systems were targeted as key settings to prevent medical errors and increase the quality of patient care. In contrast, such initiatives have faltered in ambulatory care settings, perhaps assuming these environments are safer [2]. However, a systematic review estimated that 4% of safety-related incidents in ambulatory care settings result in severe harm to patients, including death [3]. Considering most healthcare is delivered in ambulatory settings [4], with increasingly complex treatments offered, further patient safety research is needed [5].

The Joint Commission describes *safety culture* as a healthcare organization’s values, commitment, competencies, and actions in pursuit of patient safety [1]. Validated safety culture surveys exist for patients and providers in hospitals, pharmacies, nursing homes, and medical offices [6, 7]. But patient safety survey implementation has lagged within academic settings [8], including programs that provide training in spinal manipulative therapy (SMT). Similar to all healthcare specialties, professions offering SMT, most commonly chiropractic, physical therapy, osteopathy and naturopathy, often lack transparent patient safety cultures [9, 10]. Competing narratives within these professions fuel the ongoing debates about the risks and benefits of SMT [11], which may have stalled broader efforts to investigate and improve patient safety culture and performance in clinical practice and educational settings.

Nevertheless, SMT remains a widely used intervention, with about half of the adults in the U.S. receiving SMT at some point in their lives [12], usually in ambulatory care settings [13]. Few studies have considered patient safety within SMT-training programs [14, 15], even though evidence suggests positive attitudes toward patient safety require inculcation at the beginning of a healthcare worker’s career [16, 17]. Currently, the extent to which such teaching occurs in chiropractic programs is unknown. This is not surprising since published graduation standards of international chiropractic councils of education (CCEs) demonstrate few patient safety-related competencies [18, 19]. Thus, the overall purpose of our study was to evaluate patient safety attitudes of clinic stakeholders in 5 international chiropractic teaching programs. Our specific aims were to: 1) describe patient safety attitudes of chiropractic teaching clinic stakeholders 2) link key qualitative domains to quantitative survey dimensions, and 3) compare the results with those previously collected from community-based chiropractic providers [20] and publicly available from academic medical programs.

## Methods

### Study design

We used a mixed-methods, cross-sectional survey design with quantitative priority model [21]. We used the “Survey to Support Quality Improvement” [9], which was modified from the Agency for Healthcare Research and Quality (AHRQ) Medical Offices Survey for Patient Safety Culture [22] by the SafetyNET team. SafetyNET is an international and multidisciplinary research team, whose primary goal is to support strategies that promote a patient safety culture among SMT providers [9]. The content-validated survey measures 13 dimensions of patient safety attitudes [20]. For construct validity, the reliability of the AHRQ data ranged from Cronbach’s α of 0.75 to 0.90. Open-ended comments queried respondents about additional patient safety concerns. Over 7000 manual therapists internationally, including community-based chiropractors and physiotherapists (providers), have been invited to complete the SafetyNET survey with approximately 1375 respondents [20, 23].

### Comparison groups

We invited all students (*n* = 1150), faculty (*n* = 161), and staff (*n* = 113) at 7 teaching clinics associated with 5 international chiropractic programs (*n* = 1424) to participate. Four programs were located throughout North America and one in Europe; each sought participation when they had a site investigator willing to lead the project and had administrative support to conduct this study. The teaching programs and individual participants were offered anonymity regarding their involvement in the study as part of the administrative approval and informed consent processes.

We compared survey responses from the chiropractic teaching clinics to two databases. The first was of the Canadian community-based providers from four provinces of which there were 301 respondents from the 4905 invited members from their respective provincial associations [20]. The second database was the AHRQ’s 2016 User Comparative Database Report for the Medical Office Survey [24], completed by 1837 respondents, including healthcare professionals and office staff, from 137 university/medical school/academic medical programs between 2013 and 2015 [22]. Medical students were not included in the AHRQ database for direct comparison to chiropractic student interns.

### Data management

Data were collected between October 2014 and October 2016, with sites completing the survey within 6 weeks. Surveys were collected electronically (*n* = 392) and on paper (*n* = 1032) depending on program preference (2 paper, 2 electronic, 1 both paper/electronic surveys). Electronic surveys were completed on REDCap (Research Electronic Data Capture, Vanderbilt University, Nashville, TN, USA, hosted at University of Alberta and Parker University) [25]. Paper surveys were double-entered manually into the REDCap web application. The lead investigator exported datasets in .csv format and stored on a secure cloud server shared with an independent data management consultant who cleaned the data (i.e., ensured variable consistency among surveys, double-checked nonsensical entries, etc.) and created datasets for analysis.

### Data analysis

Quantitative analyses were completed with Stata 13 Software (StataCorp, College Station, TX, USA). Positive composite means were calculated for quantitative patient safety dimensions by averaging the percent positive responses on all items within each dimension. Disagreement with negatively worded items was considered a positive response. Positive percentage composite scores from the chiropractic programs were compared to the AHRQ surveys of academic medical programs and community-based SMT providers.

A multidisciplinary team conducted the independent qualitative content analysis. Four members (KP, MF, MH, SS), each representing one of the four larger programs involved in the project, provided programmatic and cultural context within their areas of expertise. Each research team member individually coded open-ended responses, in small batches of 10 randomly selected participants. The team then met via conference call to define thematic categories, review coding, and reach consensus on each category and resolve any disagreements. One member (MH) served as scribe, inputting coding decisions into a master database using NVivo® software (QSR International Pty Ltd., Victoria, Australia). The senior author (SM), an expert in qualitative analyses, reviewed all coding decisions and addressed discrepancies for the thematic categories and definitions. The full team conducted the convergent analysis, linking qualitative themes to quantitative survey dimensions. Supplementary Material #1 outlines the operational definitions for quantitative survey items and crosswalks these domains to the qualitative coding structure. We used representative quotes with identity numbers as examples of the variety of stakeholder’s patient safety perceptions from each of the different chiropractic programs/cultures.

## Results

### Respondents

The overall response rate was 45.3% (*n* = 645). Table 1 displays response rates by program and role. Slightly more interns (53.6%) and clinicians (55.9%) identified as male, while administrative/clinic staff identified as female (71.6%). Paper-based surveys (*n* = 486, 47.1%) offered higher response rates than the electronic version (*n* = 159, 40.6%). Seventy-seven respondents (11.9%) provided a comment for inclusion in the qualitative analysis.

**Table 1:** Response rates and gender of respondents by clinical roles at the five chiropractic teaching institutions

	Students (*n* = 1150)	Clinicians (*n* = 161)	Administrative/Clinic Staff (*n* = 113)	Qualitative Responses (*n* = 1424)
**Response Rates (n, %)**	469, 40.8%	95, 59.0%	81, 71.7%	77, 11.9%
**Site A^**	39.2% / 8.5%	25.0% / 3.2%	62.5% / 12.3%	13.2% / 9.1%
**Site B^**	36.4% / 51.0%	71.9% / 48.4%	90.2% / 67.9%	10.3% / 45.5%
**Site C^**	21.7% / 4.3%	35.0% / 7.4%	25.0% / 2.5%	12.6% / 15.6%
**Site D^**	41.4% / 16.4%	58.3% / 14.7%	41.7% / 6.2%	11.1% / 3.9%
**Site E^**	81.6% / 19.8%	61.0% / 26.3%	56.3% / 11.1%	13.8% / 23.4%

**^ -** (% by institution / % of the overall response)

### Quantitative comparison of patient safety dimensions

**Fig. 1:**
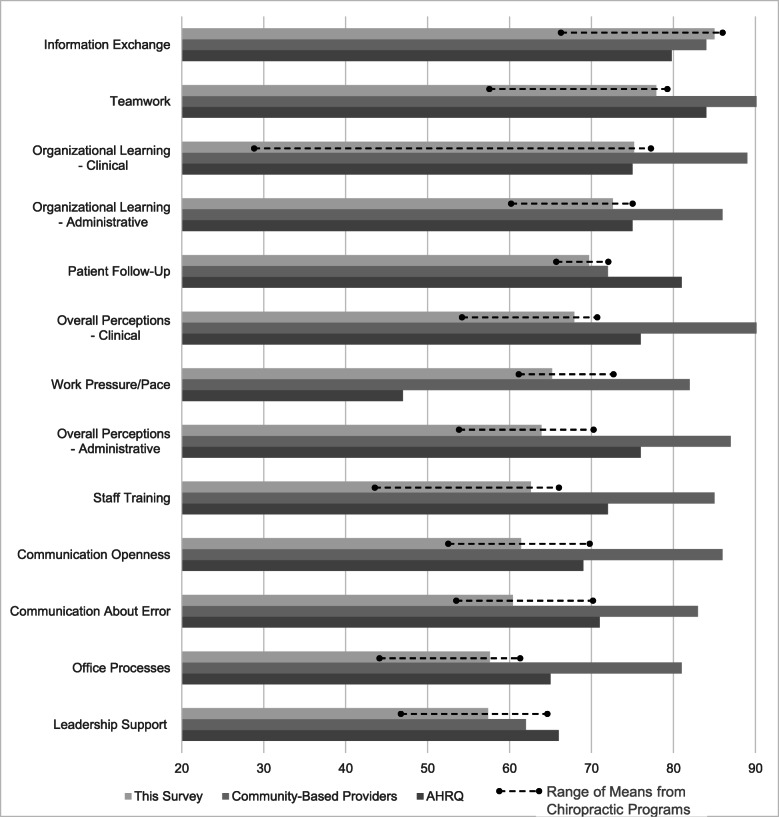
Comparison of this survey and AHRQ quantitative patient safety dimensions arranged from the largest dimension score in this survey to the least with respective ranges displayed for the 5 chiropractic programs

In our sample, the calculated Cronbach’s α for the quantitative survey items ranged from 0.62 to 0.81. Figure 1 compares the survey results of the chiropractic programs to the composite mean of the Canadian community-based providers and academic medical program (AHRQ) benchmarks for each quantitative patient safety dimension (underlined). The community-based providers and the AHRQ samples reported higher percentages of positive responses on most dimensions, with the exceptions of Information Exchange, Organizational Learning - Clinical, and Work Pressure/Pace. All three comparison groups averaged above a mean of 70% for Information Exchange, Teamwork, and Organizational Learning - Clinical/Administrative, with Information Exchange being the only dimension in which all the chiropractic programs had scored at or above 70%. Quantitative scores for both the teaching environments were lowest for Office Processes & Standardizations and Leadership Support, with average scores below 65%. Chiropractic programs reported gaps of greater than 10% compared to those from the community-based providers and medical programs for Communication About Error and Overall Perceptions - Administrative. Patient Care Tracking/Follow-Up had a gap greater than 10% for both the community-based providers and teaching clinics compared with AHRQ. While most dimension scores ranged within 15% of each other for chiropractic programs, Organizational Learning - Clinical reported the largest range (28–77%) and Patient Care Tracking/Follow-Up the smallest (66–72%). As summary dimensions, mean score and ranges for Administrative (64%, range 54–70%) and Clinical (68%, range 55–71%) dimensions of Overall Perceptions of Patient Safety and Quality were similar among all chiropractic programs, but well below the community-based provider and medical program scores.

### Qualitative analysis of chiropractic stakeholder responses

**Fig. 2:**
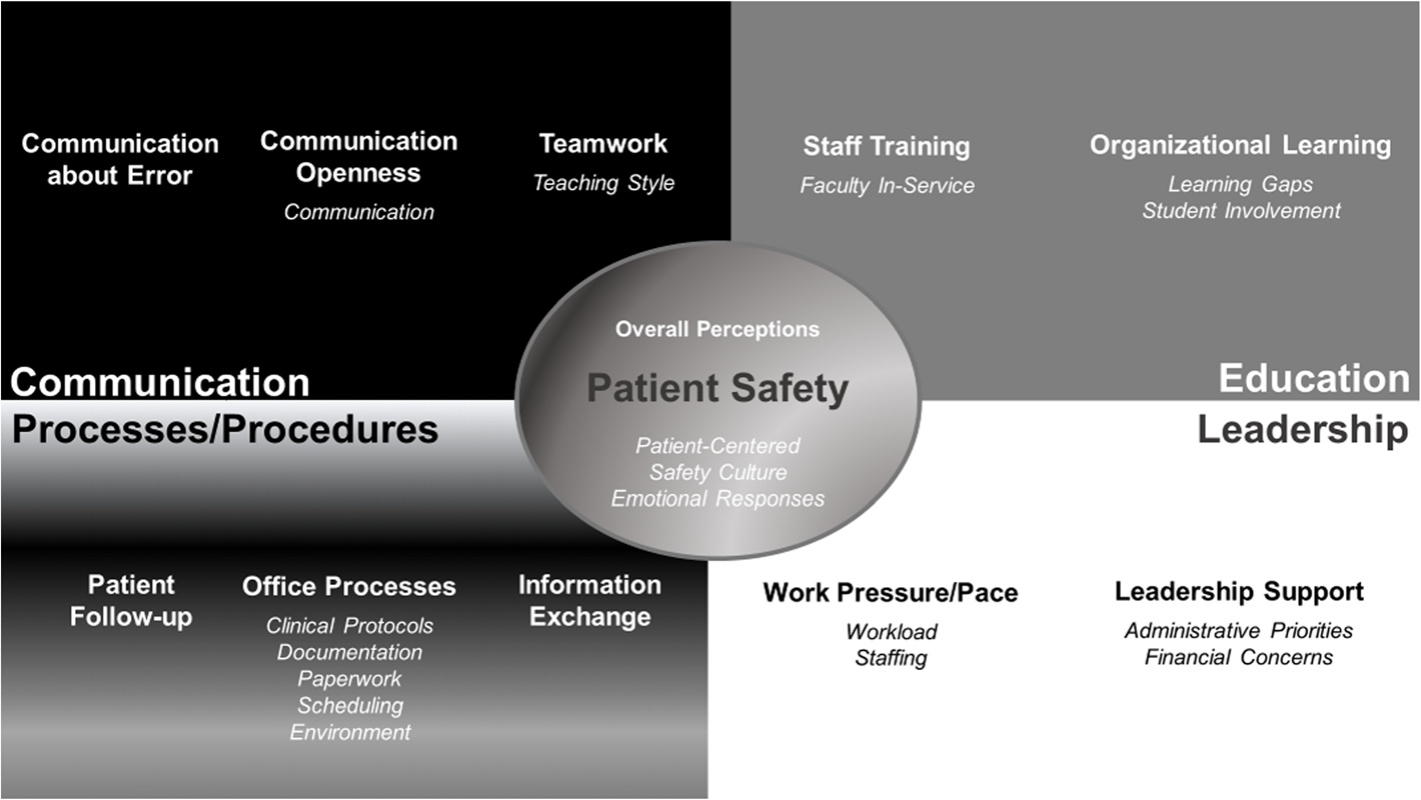
Unique emerging domains with respective quantitative dimension and qualitative themes

Figure 2 depicts the themes that emerged from our qualitative analysis of stakeholder perceptions of patient safety in chiropractic teaching clinics. Seventeen themes clustered into five domains, including Patient Safety (three themes), Communication (two themes), Education (three themes), Processes/Procedures (five themes), and Leadership (four themes). Our convergent analysis cross-walked the qualitative themes to 8 of the 11 AHRQ quantitative dimensions (Figs. 1 and 2), with no qualitative themes identified for the dimensions of Communication about Error, Patient Follow-up, and Information Exchange.

In Fig. 2, the central circle (gray gradient color) depicts the qualitative domain, **Patient Safety***,* which we related to the quantitative dimension, Overall Perceptions, and qualitative themes, *Patient-Centered, Safety Culture*, and *Emotional Responses*. For *Patient-Centered*, respondents placed patient needs before organizational priorities: “Quality should generally overrule quantity. Patient should be first priority” {Clinician69:SiteB} and “When an intern is treating a patient, the patient CARE should come first” {Intern76:SiteA}. *Safety Culture* was “improving, but there is some way to go in terms of ensuring there are robust processes in place to improve clinical practices and patient experiences” {Staff1:SiteE}. Participants noted “variations within staff perceptions” {Intern12:SiteE} ranging from “everything is well controlled and it is very difficult to make mistakes” {Intern14:SiteE} to “clinic has issues that can be resolved … we all have to pull together to make it work” {Staff66:SiteB}. The latter two themes received the most mentions from respondents and were interwoven among both quantitative dimensions and qualitative domains. Written statements demonstrated many *Emotional Responses,* with respondents offering poignant examples of missed opportunities to enhance patient safety. Specific emotions, most of which offered negative connotations (such as fear, embarrassment, anger, empathy, or burnout), were named, with capitalization and punctuation stressing respondent concerns, such as, “It is very difficult to keep up with customer care when you are under pressure!!!” {Staff27:SiteE}.

The **Communication** domain (Fig. 2, black box) included the *Communication* theme which related to Communication Openness, *Teaching Style* theme which related to the Teamwork dimension, and the Communication about Error (60%), a low scoring survey dimension, not mentioned by participants. Within *Communication*, some staff felt “voiceless” {Staff64:SiteB} or that “all voices need to be heard” {Staff65:SiteB}. Suggestions to improve communication patterns included timely responses to emails {Clinician37:SiteB}, direct communication from faculty and supervisors, and administrators being open to ideas for improving office processes and encouraging staff to express disagreement. *Teaching Style* was viewed as essential components for an effective program, with both negative and positive examples offered: “The teamwork needed to have an office run effectively and efficiently isn’t present” {Staff64:SiteB} and “[Teacher] ensures we follow-up when any changes in patient presentation occurs” {Intern6:SiteE}.

Within **Education** (Fig. 2, gray box), *Faculty In-Service* was related to Staff Training, a quantitative dimension with low mean scores (62%) among chiropractic programs; while *Learning Gaps* and *Student Involvement* were related to the Organizational Learning dimension*. Faculty In-Service* recommended: “I would love to see more communication and training for the clinicians” {Clinician70:SiteB}. In addition, interns noted how *Learning Gaps* could impact patient safety: “I received more training about procedures and policy when I worked at [ice cream store] in high school” {Intern35:SiteB}. However, *Student Involvement* in patient safety was limited as teaching programs might “not encourage any opinion from interns because they are ‘only students’” {Intern31:SiteD}.

**Processes/Procedures**, a qualitative domain (Fig. 2, gray gradient box), was related to quantitative dimensions with lower survey scores in chiropractic programs, namely, Office Processes (57%) and Patient Follow-up (69%). While no qualitative themes were connected to the Patient Follow-up or Information Exchange dimensions, Office Processes related with 5 qualitative themes: Clinical Protocols, Documentation, Paperwork, Scheduling, and *Environment*. Potential reasons for lower scores emerged from frequent respondent comments about *Paperwork:* “I struggle with [electronic health records] and paperwork requirements that interfere with ability to be fully present for patient care” {Intern35:SiteB}. Another student wrote: “Clinic seems to lose a lot of our paperwork and should be held accountable for documents they misplace” {Intern48:SiteB}. Personnel also identified *Scheduling* issues: “Too many appointments are scheduled, not allowing patients to receive enough time with their doc [tor]/intern as they need” {Staff57:SiteB}. Others mentioned outdated items in their *Environment* that might harm patients: “Equipment is in disrepair and poses a viable risk to patient safety. Moreso than hands on treatment” {Clinician29:SiteD}.

**Leadership** (Fig. 2, white box) related to the quantitative dimensions of Work Pressure/Pace and Leadership Support, with dimensions scores differing among samples (Fig. 1). Work Pressure/Pace scores suggested fewer workload issues in the chiropractic sample (65%), while Leadership Support (57%) scores compared with community-based providers and medical programs identified area of improvement. Four themes emerged from the Work Pressure/Pace dimension: *Workload* and *Staffing*. One intern noted: ‘not enough time to do all the treatments, especially if a patient was talkative and clinicians aren’t readily available” {Intern21:SiteE} and “double booking interns and booking too many patients per hour has become a constant issue despite procedures in place to prevent this from happening” {Clinician33:SiteC}. Leadership support themes identified were: *Administrative Priorities* and *Financial Concerns.* Participant supporting comments included: “Administrators make decisions based on cheapest option available, not based on patient care and student learning” {Intern25:SiteC} and “… very poor and unreasonable leadership … my frustrations in clinic have come directly from that” {Intern34:SiteD}.

## Discussion

Our study expands on the previous work by SafetyNet investigators to enhance patient safety culture among providers of spinal manipulation therapy [9, 20]. To our knowledge, our study is the first to evaluate patient safety attitudes among stakeholders in chiropractic teaching clinics and compare these to academic medical programs and community-based clinicians offering SMT. The majority of U.S. medical schools include some level of training in patient safety [26, 27]. However, less is known about the amount or content of training in patient safety offered to chiropractic students, although this topic is an encouraged addition to the accreditation process for chiropractic programs [18, 19]. In comparison to medical students, chiropractic students may be disadvantaged in learning about patient safety as much of their clinical training occurs in monodisciplinary clinical settings instead of interdisciplinary healthcare environments [13]. Chiropractic students may benefit from participating in interprofessional educational programs about patient safety as have students in other health disciplines [26, 28]. As evident in other healthcare environments [29], such interprofessional programs may alleviate the competing tensions between professions providing SMT and improve collaboration in advancing patient safety [11].

While direct comparison between these academic programs is limited due to the lack of medical student data in the AHRQ database, our findings highlight the need for continued focus on patient safety training for chiropractic students and clinicians alike. Encouragingly, both academic and clinical settings scored relatively highly (means 72–85%) on AHRQ survey domains for Information Exchange (record-sharing), Teamwork (close working relationships between staff and providers), and Organizational Learning (culture supporting changes in office processes). However, these domains focus less on patient-based interactions and more on how clinic stakeholders interrelate with one another or with those in other clinics. Somewhat surprisingly, *Patient-Centered* was a recurrent theme interwoven throughout the qualitative feedback provided by chiropractic stakeholders. Despite recognition of patient-centeredness as the foundation for quality of patient care [30–32], it is often overlooked in patient safety surveys [22, 33–35]. A recent pilot project assessing patient-centered attitudes conducted at some of the same chiropractic programs included in our study, revealed a lesser focus on patient-centered care than similar studies with medical students [36, 37]. This suggests chiropractic curricular content (and hidden/null curriculum content) likely influences students’ attitudes toward patient-centered care.

For most AHRQ survey domains, the chiropractic programs had significant gaps compared with the Canadian community-based providers and the medical academic programs in patient safety attitudes, with qualitative findings from the chiropractic sample accentuating specific areas for improvement. For example, low quantitative scores for Office Processes may indicate these teaching clinics have inefficient clinic procedures to organize workflow or check accuracy of work performed. Qualitative feedback identified challenges of securing patient data, creating logical electronic health record forms, minimizing paperwork, and efficiently scheduling patient appointments and follow-up care. These findings are echoed in other studies focused on electronic health records [38–40]. Additional explanations for these gaps need further exploration, but may be explained by the initial focus of the patient safety culture movement encouraged on the medical systems, due to the higher prevalence of severe patient conditions, shorter visit times affecting patient-provider relationships, more funds available to direct toward this initiative, and improved organizational structure/employees understanding of safety culture within most medical systems allowing for less fear of consequences for staff who identify/report errors [1, 2].

Our analysis also identified an important gap in the current AHRQ Medical Offices Survey. We propose adding questions related to *Environment*. While maintaining the physical environment and equipment is a critical responsibility for healthcare institutions [41, 42], teaching clinics have added challenges. Healthcare students might not recognize safety risks in the environment or understand how to use equipment properly, leading to potential harms to patients [43]. Novel strategies, such as safety huddles, might be incorporated into clinical training experiences to enhance the identification of potential threats to patient safety, including those arising from equipment and clinical setting factors [44].

Communication, another key to successful patient safety movement, emerged as a problematic domain in our study. In all three settings, Communication Openness (expression of alternative viewpoints) and Communication about Error (medical error reporting) had quantitative scores just over 60%, but little-to-no qualitative feedback, particularly around error reporting. While several reports indicate the need for better communication, how to best put this into practice is yet to be discovered [1, 45]. Within medical programs, student knowledge of key patient safety initiatives and the latest evidence on reducing, reporting, and correcting medical errors is inconsistent [27, 46]. Further research is needed in how healthcare professional students learn how to recognize errors and follow-up when identified, as well as specifically exploring communication openness to assess if this is an area of need.

Lastly, participants discussed the largely negative impacts of Leadership Support on patient safety. Although the quantitative scores of chiropractic stakeholders suggest fewer Work Pressure/Pace issues than academic medical settings, several qualitative themes emerged including issues with *Workload* and *Staffing*. Heavy workload concerns may lead directly to medical errors, but how poor staffing or overbooking patients impacts student training is less well understood. Similarly, ‘Leadership Support’ qualitative themes (*Administrative Priorities* and *Financial Concerns*) provide insights and awareness of what could be improved. Leadership Support in organizations with robust patient safety cultures have been found to have standardized behavioral expectations that are known and modelled by all in the organization [6]. Therefore, it is important for leaders in health care organizations to make a commitment to establishing and sustaining a patient safety culture [47].

### Strengths, limitations, and future directions

Study strengths include the international context of chiropractic teaching clinics, a mixed methods design providing explanatory insights of survey scores, the inclusion of chiropractic students/interns’ unique perspectives on patient safety, and the innovative comparison to academic medical programs using the AHRQ Medical Offices Survey and previous research with manual therapy clinicians working in community-based settings. Limitations include the lack of student representation in the medical program comparative database, which has direct implications on the validity of the comparison. The internal consistency of our scale was lower than the AHRQ survey but still good; however, 7 items were identified as questionable and needs further exploration in future studies. Additionally, there was low survey response rate in some chiropractic programs and only 11.9% of survey respondents offered feedback to the open-ended question. The limited response rate might be due to survey burden, available time, or lack of opinions about the topic. However, we cannot rule out that respondents opted to not complete this section of the survey due to concerns about their privacy or anonymity, fear of retaliation from their employers should their responses become known, feelings of powerlessness in addressing the problem of patient safety, or lack of knowledge about the topics under discussion. Thus, our findings should be considered exploratory rather than definitive in regards to stakeholders’ perceptions of patient safety in chiropractic teaching clinics.

A limitation of using a single-item open-ended question to collect qualitative feedback was that survey respondents tended to emphasize potential problems in patient safety in lieu of offering examples of what chiropractic education programs might be doing right. Future efforts will ask respondents to provide information on both best practices and opportunities for improvement. Similarly, while brief open-ended questions are useful in mixed methods research, additional insights might be gained from ethnographic field methods or in-depth interviews from the various stakeholders. For example, chiropractic teaching clinics had wide variations on quantitative scores related to *Organizational Learning*; however, the lack of qualitative feedback prevents us from offering a discussion on this topic. In contrast, a larger-scale qualitative study could target programs with high and low scores to conduct in-depth explorations stakeholders’ insights on how their organization learns from past mistakes or introduces new practices into clinic settings to improve patient safety.

Most importantly, future patient safety studies within the health professions offering SMT should include patients’ participation. Few studies have evaluated patients’ values, fears, and expectations related to SMT [48–52]. Finally, teaching clinics – both chiropractic and medical - might also benefit from additional exploratory research to better understand how administrative priorities, communication styles, training program, and clinical protocols impact patient safety culture within these unique ambulatory care settings.

## Conclusion

Clinic stakeholders identified multiple areas for improvement in patient safety within chiropractic educational programs. Teamwork and information exchange were considered strengths in these settings. Respondents emphasized the need for patient-centered administrative priorities, improved work pressure/pace, standardized office processes, and enhanced communication about patient care between clinic stakeholders. Student feedback, although minimized by some respondent groups, articulated the emotional side of missed opportunities in patient safety and suggested key areas for additional training for trainees and faculty alike.

## Supplementary information

**Additional file 1.** Patient safety operational definitions and representative quotes for qualitative themes.

## Data Availability

Data available upon request include responses from dimension questions and respondent’s role. Identification of respondent’s institution will not be available per respective data agreements. Data requests can be made to corresponding author (ORCID iD: 0000–0002-1536-112X). Reuse of data is not permitted without authorization from the authorship team.

## Abbreviations

AHRQ: Agency for Healthcare Research and Quality
CCEs: Chiropractic councils of education
IOM: Institute of Medicine
REDCap: Research Electronic Data Capture
SafetyNET: An international and multidisciplinary research team, whose primary goal is to support strategies that promote a patient safety culture among SMT providers
SMT: Spinal manipulative therapy
U.S.: UNITED States
