# Supplementary material for: Patient safety in chiropractic teaching programs: a mixed methods study
Source: Chiropr Man Therap. 2020 Sep 18;28:50. doi: 10.1186/s12998-020-00339-0 (PMC7500017; doi:10.1186/s12998-020-00339-0)
Supplement: Supplementary file 1 — Additional file 1. Patient safety operational definitions and representative quotes for qualitative themes. [file 12998_2020_339_MOESM1_ESM.docx]

**SUPPLEMENTAL MATERIAL.** Patient safety operational definitions and representative quotes for qualitative themes.

| **Domain** | **AHRQ Survey**  **Quantitative Dimensions^18^** | **Qualitative Theme Definitions** | **Representative Quotes** |
| --- | --- | --- | --- |
| **PATIENT SAFETY** | **Overall Perceptions:**  The quality of patient care is more important than getting more work done, office processes are good at preventing mistakes, and mistakes do not happen more than they should.  *Includes two AHRQ patient safety dimensions: Overall Perception – Clinical and Overall Perception - Administrative | ***None*** | - No Comments |
|  | **None** | ***Patient-Centeredness:***  Value or attitude that the patient is the important person in doctor-patient relationship; care should focus on patient needs. | - When an intern is treating a patient, the patient CARE should come first. {Intern76:SiteA} - Quality should generally overrule quantity. Patient should be 1^st^ priority. {Clinician69:SiteB} - The need for volume outweighs evidence-based care; wellness care 2x a week. {Clinician67:SiteB} - I feel as if patients are under-cared for because we are too worried about what is medically warranted. Why can’t we just do what IS warranted? {Intern39:SiteA} - I have come into contact with several interns and doctors who don’t care about the patients’ quality of life and care. For some interns, this is just a joke to them. They don’t take it seriously and don’t find new ways of treatment for their patients…We need more patient care focus. {Staff36:SiteB} |
|  |  | ***Safety Culture:***  Overriding attitude (proactive or reactive) toward patient and staff safety concerns shared by clinic members. | - Everything is well controlled and it is very difficult to make mistakes. Clinic is well managed in all areas and we are doing audits every year to control and improve mistakes. {Intern14:SiteE} - The clinic has issues that can be resolved. No one likes change, but sometimes change has to happen to move forward. All voices need to be heard. {Staff66:SiteB} |
|  |  | ***Emotions:***  Emotions or feelings described in relation to patient safety: fear, distress, condescension, motivation, encouragement, guilt, confidence, frustration, empathy, anger, burnout. | - It is very difficult to keep up with customer care when you are under pressure!!! {Staff27:SiteE} - [Teachers] sometimes display a condescending tone when discussing a case which can be quite distressing. It would be nice to get a good vibe from all [teachers] – motivation and encouragement – rather than leaving us with a feeling of guilt. {Intern28:SiteE} - I feel tired, overworked, and with added pressure to [provide] excellent care even though I have way too many patients at once. {Intern29:SiteE} - It’s embarrassing when equipment is non-functional. {Intern24:SiteB} |
| **COMMUNICATION** | **Communication about Error:**  Providers in the office are open to staff ideas about how to improve office processes, and staff are encouraged to express alternative viewpoints and do not find it difficult to voice disagreement. | ***None*** | - No Comments |
|  | **Communication Openness:**  Providers in office are open to staff ideas about how to improve office processes, and staff are encouraged to express alternative viewpoints and do not find it difficult to voice disagreement. | ***Communication:***  All ways of transferring information from one group to another. | - Faculty clinicians, especially new hires, are petrified to ask questions because they are reminded about being on probation…Emails to admin[istration] are never responded to in a timely basis. Sometimes taking weeks. {Clinician37:SiteB} - Doctors have NO respect for the staff – confronting staff members at the front desk in front of the patients and the students...Communication between staff comes from other staff rather than your immediate supervisor. {Staff65:SiteB} - The staff feels ‘voiceless’. Conversations with your supervisor end with “that’s a battle that I don’t want to fight” or “my hands are tied” or just a shrug of the shoulders. {Staff64:SiteB} |
|  | **Teamwork:**  Office has a culture of teamwork, mutual respect, and close working relationships among staff and providers. | ***Teaching Style:***  Personal teaching style; teacher attitudes, demeanor, or actions toward students. | - [Teacher] ensures we follow-up when any changes in patient presentation occurs or if they are not responding well to treatment. We go over contraindications to SMT [spinal manipulative therapy] and to certain modalities and evaluate whether these apply to the patient. {Intern6:SiteE} - Disagreements between [teachers] upon type of care to be provided. {Intern5:SiteE} - We are treated more as children here rather than adults, much less DOCTORS. I am, however, pleased that our staff doctors are supportive and enlightening toward our educative endeavors. {Intern39:SiteA} - I feel the co-managing confuses patients and often makes them feel uneasy. I feel it negatively effects the patient-practitioner encounter. {Intern8:SiteE} |

| **EDUCATION** | **Staff Training:**  The office gives providers and staff effective on-the-job training, trains them on new processes, and does not assign tasks they have not been trained to perform. | ***Faculty In-Service:***  Ongoing training for faculty, in-services on important topics, other educational meetings. | - I would love to see more communication and training for the clinicians. {Clinician70:SiteB} - Clinic needs to all be one the same page when we update our ICD-10 codes. [Insurance] paperwork have been a headache even with multiple meetings, all the docs don’t know what needs to be done. {Intern52:SiteB} |
| --- | --- | --- | --- |
|  | **Organizational Learning:**  The office has a learning culture that facilitates making changes in office processes to improve the quality of patient care and evaluates changes for effectiveness.  *Includes two AHRQ patient safety dimensions: Organizational Learning – Clinical and Organizational Learning - Administrative | ***Learning Gaps:***  Student perceptions of teaching and teachers' actions that contribute negatively to professional practice and pose potential safety issues. | - Need more case / re-presentation [follow-up] slots for interns. {Intern4:SiteE} - Never any proper formal training on different modality machines we use. {Intern18:SiteD} - For me personally, I struggle with EHR & paperwork and requirements that it interferes with ability to be fully present for patient care. I think I've received more training about procedures and policy when I worked @ [ice cream store] in high school. I get that some people are naturals or confident enough that they don't require much guidance, but I think clinicians could be better about determining who could benefit from more guidance instead of spending the most time giving patients to only the best and helping them be better. {Intern35:SiteB} - Incoming interns are ill-prepared to see outpatients and require close supervision. Considering the required number of patient visits - especially combined with the number of patients that have insurances that require the clinician to treat them - there is a potential for patient errors during the early part of internship. {Clinician38:SiteB} - I feel that since coming into this clinic my time and efforts have been used to not 'stomp on any toes' rather than actually learning proper basic procedures needed to be able to set-up an office upon graduation. This clinic is pumping out thousands of associates and not chiropractors. {Intern40:SiteA} |
|  |  | ***Student Involvement:***  Role of interns in clinic; ability to participate in leadership roles in developing clinic processes or procedures. | - The understanding and commitment of our clinic interns is variable and difficult to estimate. {Clinician26:SiteE} - [Admin] does not encourage any opinion from interns because they are ‘only students’. {Intern31:SiteD} |

|  | **Patient Follow-up:**  The office reminds patients about appointments, documents how well patients follow treatment plans, follows up with patients who need monitoring, and follows up when reports from an outside provider are not received. | ***None*** | - No Comments |
| --- | --- | --- | --- |
|  | **Office Processes:**  The office is organized, has an effective workflow, has standardized processes for completing tasks, and has good procedures for checking the accuracy of work performed. | ***Clinical Protocols:***  Protocols or procedures used in the clinic, not necessarily related to safety. | - Patient care quality is consistently negatively affected by procedure. Too much paperwork, scheduling difficulties. {Intern54:SiteB} - It is inappropriate that patient files are left at the front areas [of clinic]. Our [area] takes it back into the file room at the end of each day to be locked up. Others leave their files on the front desk. This is affecting patient confidentiality. {Intern20:SiteD} - When changes are made within the clinic, but certain areas of the clinic don’t have to adhere to the change. Scheduling is not the same in all areas…notification of appointments is not the same…the clinic needs to be one accord when it comes to scheduling, checking in and checking out patients. {Staff65:SiteB} - Things are improving but there is some way to go in terms of ensuring there are robust processes in place to improve clinical practices and patient experience. {Staff1:SiteE} |
|  |  | ***Documentation:***  Computers, electronic health records [EHR], patient files, or other systems to maintain records of patient care, student work, or administrative processes. | - Exam forms, history forms do not correlate well with EHR and takes longer to do paperwork than should be necessary. {Intern48:SiteB} - Scheduling and medical records both need to be addressed…Not finding / having a patient’s chart available can affect patient safety, but mostly affects quality of the patient visit. {Clinician33:SiteC} - EHR does not prevent interns from making mistakes with patient care. Find a better system or upgrade. {Intern47:SiteB} - Obtaining an EHR system will allow for greater patient safety and an increase in quality of care. {Intern75:SiteA} |
|  |  | ***Paperwork:***  Completion of required documentation related to patient care or student assignments. | - We need to address the amount of paperwork and protocols set in place to best fit the high volume of patients we are currently seeing. {Intern29:SiteE} - Clinic seems to lose a lot of our paperwork and should be held accountable for documents they misplace. {Intern48:SiteB} - [Clinic rotations] would benefit from having similar rules / ways of completing paperwork…docs [doctors] do not have time to complete notes, and so when a new intern receives a note, an [re-evaluation exam] was repeated. [This] has happened twice. {Intern34:SiteB} |
|  |  | ***Scheduling:***  Process for scheduling patient appointments in clinic or for diagnostic procedures. | - Double booking interns and booking too many patients per hour (not having a second student available for the room) has become a constant issue despite procedures in place to present this from happening. {Clinician33:SiteC} - Sometimes I think too many appointments are scheduled not allowing patient to receive enough time with their doctor/intern as they need. Patient feels treatment is rush. {Staff57:SiteB} - Not being able to schedule patients when I need to (overbooked, clinician hours different from patient hours). {Intern54:SiteB} |
|  |  | ***Environment:***  Durable medical equipment (tables, imaging), materials, supplies required for the delivery of healthcare and physical environment or setting of the clinic (rooms, waiting area, décor). | - Our tables and drop pieces are not properly maintained which causes them to stick during adjustments. {Intern18:SiteD} - Equipment is in disrepair and poses a viable risk to patient safety. Moreso than hands on treatment. {Clinician30:SiteD} - We need new tables. Some of the tables are flexion distraction tables and they are not the most stable especially when it comes to the rotation piece. Some tables do not even more up and down which is not helpful when trying to administer care. {Clinician33:SiteC} - Broken seals/locks on tables that have never been fixed create fall risk for patients. {Intern53:SiteB] - We need to make sure all equipment is working. We have 2 broken tables for the last 2 [terms]. {Intern45:SiteB} - Patient safety is lacking here, from patients hitting their heads on paper towel dispensers to patients breaking their legs on carpets to patients being electrocuted with IFC. {Intern19:SiteD} - We have not had air conditioning for over a month. There’s a fan in the hallway instead. Many people including children walk by. They can potentially put their fingers in the blades and get chopped off. {Clinician68:SiteB} - Problems with carpet could cause tripping hazard. {Staff59:SiteB} |
|  | **Information Exchange:**  The office exchanges information accurately, completely, and in a timely fashion. | ***None*** | - No Comments |

| **LEADERSHIP** | **Work Pressure / Pace:**  There are enough staff and providers to handle the patient load, and the office work pace is not hectic. | ***Workload:***  Perceptions regarding number of patients or amount of work to be completed during assigned work shift. | - Our clinic is currently taking on more patients than it can handle. We are breaking records in terms of numbers (patient and income) but at the expense of reducing our quality of care. Interns are over working spending entire days just to keep up with the demanding workload. {Intern29:SiteE} - Double booking interns and booking too many patients per hour (not having a second student available for the room) has become a constant issue despite procedures being in place to prevent this from happening. {Clinician33:SiteC} - Overloading the doctor’s schedules can only give the faculty a short time with patients. Not sure this gives the patient the best care. See a lot of the same patient weekly – do they need to come every week? Is it in their care plan? Do they even get a care plan? {Staff60:SiteB} - There should be a cap on how many patients a doctor sees a day. Patient care is not fully or accurately executed because of “the quicker you get them in the quicker you get them out” because another patient needs the room. {Staff63:SiteB} |
| --- | --- | --- | --- |
|  |  | ***Staffing:***  Non-teaching clinic *s*taffing (case managers, front office staff) and teaching personnel required to adequately staff the clinic to meet needs of patients and interns. | - Few case managers compared to patient load. Results that patient has to wait a couple days before getting treatment for their complaint. {Intern3S:SiteE} - There is not enough staff in the clinic to cope with the amount of patients and interns and extra work. There is also too much admin work to do on the reception desk causing us to make constant errors. {Staff27:SiteE} - Number of times there has been no [teachers] available to see patients. {Intern13:SiteE} - Need to evenly distribute patients throughout [clinic]. [One area] is slow in patients because of all the clinician changes and [another area] has way too many [patients]. {Intern46:SiteB} - [Teacher] on floor duty should try to work more efficiently for the benefit of all the interns working rather than taking a long time on one case if there is a queue. {Intern6:SiteE} |
|  | **Leadership Support:**  Office leadership actively supports quality and patient safety, places a high priority on improving patient care processes, does not overlook mistakes, and makes decisions based on what is best for patients. | ***Administrative Priorities:***  Goals or focal points of organizational leadership. | - In my opinion a lot of the clinical/college processes are driven too much by politics and not the students’ interest of being able to think freely. {Intern10:SiteE} - Our clinic is great, and the environment down there is great as well. I believe there is very poor and unreasonable leadership from [program] however, and all of my frustrations in clinic have come directly from that. {Intern24:SiteD} - All [administrators] do is sit behind their own desks and offer little support for the interns to meet their numbers and improve clinic functioning. {Intern31:SiteD} - There is a lack of leadership [at program]. I have witnessed very unprofessional behavior among the administrative team and it trickles down to staff and faculty & unfortunately students. {Clinician37:SiteB} - [Administrator’s name] needs a lesson in management. Treats staff very disrespectful. Sits in office all day playing on computer, entering contests. Won’t work or help staff at all. {Staff61:SiteB} - The administration here doesn’t seem to care at all. Get someone full-time for marketing. Preferable someone who has a background in marketing. {Intern77:SiteA} - [Clinic] should be at forefront of patient recruitment and be involved in community and right now [clinic] is very separated from community. {Intern19:SiteD} - With the lack of patients at [clinic], we feel there is no help from [program] to help encourage greater inflow of patients. {Intern31:SiteD} |
|  |  | ***Financial Concerns:***  Finance issues of clinic; treatment price; insurance-related concerns | - Administration makes decisions based on the cheapest options available, not based on patient care and student learning. {Intern25:SiteC} - Patients often complain prices are too expensive for student-based care. {Intern72:SiteA} - Had one patient leave because of way clinic handles insurance. Told one thing then another after one visit of care…angered patient into going elsewhere for care. {Intern74:SiteA} |
